# Enhanced Surveillance for Fatal Dengue-Like Acute Febrile Illness in Puerto Rico, 2010-2012

**DOI:** 10.1371/journal.pntd.0005025

**Published:** 2016-10-11

**Authors:** Kay M. Tomashek, Aidsa Rivera, Brenda Torres-Velasquez, Elizabeth A. Hunsperger, Jorge L. Munoz-Jordan, Tyler M. Sharp, Irma Rivera, Dario Sanabria, Dianna M. Blau, Renee Galloway, Jose Torres, Rosa Rodriguez, Javier Serrano, Carlos Chávez, Francisco Dávila, Janice Perez-Padilla, Esther M. Ellis, Gladys Caballero, Laura Wright, Sherif R. Zaki, Carmen Deseda, Edda Rodriguez, Harold S. Margolis

**Affiliations:** 1 Dengue Branch, Division of Vector-Borne Diseases, Centers for Disease Control and Prevention (CDC), San Juan, Puerto Rico; 2 Puerto Rico Institute of Forensic Sciences, San Juan, Puerto Rico; 3 Infectious Diseases Pathology Branch, Division of High Consequence Pathogens and Pathology, CDC, Atlanta, Georgia, United States of America; 4 Bacterial Special Pathogens Branch, Division of High Consequence Pathogens, CDC, Atlanta, Georgia, United States of America; 5 Demographic Registry of Puerto Rico, San Juan, Puerto Rico; 6 Geospatial Research, Analysis, and Services Program, Division of Toxicology and Human Health Sciences, ATSDR, Atlanta, Georgia, United States of America; 7 Puerto Rico Department of Health, San Juan, Puerto Rico; North Carolina State University, UNITED STATES

## Abstract

**Background:**

Dengue is a leading cause of morbidity throughout the tropics; however, accurate population-based estimates of mortality rates are not available.

**Methods/Principal Findings:**

We established the Enhanced Fatal Acute Febrile Illness Surveillance System (EFASS) to estimate dengue mortality rates in Puerto Rico. Healthcare professionals submitted serum and tissue specimens from patients who died from a dengue-like acute febrile illness, and death certificates were reviewed to identify additional cases. Specimens were tested for markers of dengue virus (DENV) infection by molecular, immunologic, and immunohistochemical methods, and were also tested for West Nile virus, *Leptospira spp*., and other pathogens based on histopathologic findings. Medical records were reviewed and clinical data abstracted. A total of 311 deaths were identified, of which 58 (19%) were DENV laboratory-positive. Dengue mortality rates were 1.05 per 100,000 population in 2010, 0.16 in 2011 and 0.36 in 2012. Dengue mortality was highest among adults 19–64 years and seniors ≥65 years (1.17 and 1.66 deaths per 100,000, respectively). Other pathogens identified included 34 *Leptospira spp*. cases and one case of *Burkholderia pseudomallei* and *Neisseria meningitidis*.

**Conclusions/Significance:**

EFASS showed that dengue mortality rates among adults were higher than reported for influenza, and identified a leptospirosis outbreak and index cases of melioidosis and meningitis.

## Author Summary

Dengue is a major public health problem in the tropics. Despite its global importance, population-based mortality rates attributable to dengue are largely unknown. Dengue vaccines are now in late stage clinical trials and one vaccine has been licensed in several countries. Evidence-based decisions regarding the future use of dengue vaccines will depend on robust estimates of disease burden which should include mortality. To estimate mortality due to dengue in Puerto Rico, where dengue is endemic, we developed an enhanced surveillance system to detect fatalities due to a preceeding dengue-like acute febrile illness using more sensitive case identification and laboratory methods than the previous passive method. This surveillance system found the dengue mortality rate was 1.05 per 100,000 Puerto Rico residents in 2010, the highest rate ever detected. Among adults aged 19–64 years, mortality from dengue (1.17 deaths per 100,000) was higher than from other infectious diseases, including influenza. The utility of this enhanced surveillance system was further proven through the identification of an outbreak of leptospirosis as well as detection of other diseases of public health importance, including melioidosis and meningitis.

## Introduction

Dengue is a major public health problem worldwide. While most dengue virus (DENV) infections are asymptomatic or result in a mild acute febrile illness (AFI), some are life-threatening due to plasma leakage [1, 2]. With no antivirals to treat dengue or prevent its severe manifestations [3], early recognition of severe dengue and timely supportive care is used to reduce mortality [4–6]. Several dengue vaccines are in late stage clinical trials and one was recently licensed in several countries [7]. Decisions regarding their use will depend on vaccine performance and safety, and reduction of disease burden, including deaths.

Globally, an estimated 3.9 billion people are at risk of DENV infection, and 96 million dengue cases are estimated to have occurred in 2010 alone [8]. Despite its global presense, robust estimates of population-based dengue mortality rates are lacking. Most estimates have been derived from passive surveillance data [9–14] or hospital-based, retrospective case reviews [15, 16]. During epidemic periods, these methods have produced annual mortality rates that ranged from 0.30–0.59 deaths per 100,000 population. However, these approaches have not been validated as to under recognition due to misdiagnosis or under reporting [14].

Dengue has been endemic in Puerto Rico since the late 1960s [17, 18], and after the first deaths were reported in 1986, surveillance for deaths due to dengue was established in 1987 [19]. Mortality data have been collected through the island-wide Passive Dengue Surveillance System (PDSS), a hospital-based Infection Control Nurse Dengue Surveillance System (ICNDSS) that operated until 2007, and review of death certificates. Evaluations of these systems identified misdiagnosis and underreporting of cases, and failure to include dengue on death certificates of known laboratory-positive dengue cases [14, 20]. Few suspected fatal cases had tissue specimens or appropriately timed pre-mortem serum specimens for diagnostic testing, which resulted in a high proportion of indeterminate diagnostic results [14, 19–21].

In 2009, the Centers for Disease Control and Prevention Dengue Branch (CDC-DB), Puerto Rico Department of Health (PRDH), Instituto de Ciencias Forenses de Puerto Rico (in English, Puerto Rico Institute of Forensic Sciences [PRIFS]), Demographic Registry of Puerto Rico, and CDC Infectious Diseases Pathology Branch (CDC-IDPB) established the Enhanced Fatal Acute Febrile Illness Surveillance System (EFASS) to define mortality due to dengue-like AFI, and determine the etiology of these cases. We describe the findings from the first three years of EFASS.

## Methods

### Ethics Statement

This project underwent CDC institutional review and formal institutional review board review was not required since the case-patients were deceased. Because cases were reported in the context of public health surveillance, the informed consent of patients’ families was not sought. Patient identifiers were removed from the dataset prior to analysis.

### Data Sources and Case Detection

EFASS used enhanced surveillance to detect dengue-like AFI deaths, improve reporting, and standardize collection of specimens at autopsy. While PDSS provided retrospective diagnostic data on fatal suspected dengue cases reported early in their illness, the primary source of EFASS cases was reporting by participating epidemiologists, pathologists, and registry statisticians. Specifically, they were asked to report and submit samples from all fatal cases whose death occurred during or immediately following a dengue-like AFI defined by the presence of fever (body temperature ≥38.0C axillary) or history of fever for ≤7 days. This included deaths with pre-defined diagnostic codes on the medical record, autopsy report or death certificate (S1 Appendix); the list of ICD codes was developed in 2009 after a review of the 1994–2007 fatal laboratory-positive dengue cases was conducted. Surveillance was enhanced by collaboration with pathologists and epidemiologists at hospitals most likely to encounter severe dengue cases, and included training and provision of standardized protocols. Dengue-like AFI fatalities that occurred at home or within 24 hours of hospital admission and referred to PRIFS were included. Collaborators were contacted weekly, and death certificates, PDSS, and National Notifiable Diseases Surveillance System (NNDSS) were routinely queried to identify suspected cases.

### Specimen and Data Collection

Once a suspected case was identified, serum, whole blood, and tissue specimens were obtained, and PRIFS pathologists completed a Surgical Pathology and Autopsy Report (SPAR) (S2 Appendix). Cases with history of respiratory failure had a nasopharyngeal swab submitted for testing.

Data was abstracted from medical records of all health care visits during the illness for laboratory-positive dengue cases using a standard instrument that captured demographic characteristics, past medical history, clinical course, and management.

### Diagnostic Testing

Serum specimens were tested by a DENV-serotype specific real time, reverse transcriptase-polymerase chain reaction (rRT-PCR) assay [22], an anti-DENV IgM enzyme-linked immunosorbent assay (MAC ELISA) [23], and an anti-DENV IgG ELISA [24]. Serum specimens were also tested by anti-West Nile virus (WNV) MAC-ELISA and, if positive, WNV-specific rRT-PCR and 90% plaque reduction neutralization tests (PRNT_90_) were performed [25]. Serum specimens with sufficient volume were sent to CDC Bacterial Special Pathogens Branch and tested for *Leptospira* IgM using the ELISA ImmunoDOT kit (GenBio, Inc., San Diego, CA). Acute specimens were tested for nucleic acid by rRT-PCR and 20 *Leptospira* reference antigens representing 17 serogroups by microscopic agglutination test (MAT) [26]. RNA was extracted from nasopharyngeal specimens and tested for Influenza A and B viral genome by rRT-PCR [27].

Tissue specimens were tested at the CDC-IDPB for DENV antigen or nucleic acid by immunohistochemistry (IHC) and RT-PCR, respectively [28]. If clinical presentation or histopathology were suggestive of another etiology pathogen-specific diagnostic testing was performed [26, 29].

### Definitions

A *fatal suspected dengue-like AFI case* had a dengue-like AFI that immediately preceded death in a Puerto Rico resident. A *fatal laboratory-positive dengue case* was a suspected case with DENV nucleic acid in serum or tissue; DENV antigen in tissue; IgM seroconversion in paired specimens; or IgM in a single specimen. A *fatal laboratory-negative dengue case* was a suspected case with no molecular, immunodiagnostic or IHC markers of DENV infection. A *fatal laboratory-indeterminate dengue case* was a suspected case with no DENV nucleic acid or anti-DENV IgM in the acute serum specimen (collected ≤5 days post-illness onset [DPO]) and no available convalescent serum specimen (≥6 DPO). A *fatal dengue co-infection* was a fatal suspected dengue-like AFI case with DENV nucleic acid in serum or tissue and another pathogen detected by PCR or IHC. A *primary DENV infection* was a fatal laboratory-positive dengue case without anti-DENV IgG in the acute serum specimen [30] and a *secondary DENV infection* had anti-DENV IgG in the acute specimen.

A *fatal laboratory-confirmed leptospirosis case* was a suspected dengue-like AFI case with ≥4-fold increase in MAT titers in paired specimens, MAT titer ≥800 in a single specimen, or detection of *Leptospira spp*. nucleic acid in serum by PCR or antigen in tissue by IHC. A *probable fatal leptospirosis case* had a MAT titer >200 but <800 in a serum specimen.

*Dengue fever* (DF), *dengue hemorrhagic fever* (DHF) and *dengue shock syndrome* (DSS) were defined according to the 1997 World Health Organization (WHO) guidelines [31] (Table 1). *Dengue*, *dengue with warning signs*, and *severe dengue* were defined according to 2009 WHO guidelines [2]. Definitions for severe dengue, other clinical features and medical complications are shown in Table 1.

**Table 1:** Clinical findings used to define fatal laboratory-positive dengue cases.

Clinical Syndrome or Condition	Laboratory or Clinical Definitions Used	Ref.
**Dengue fever, dengue hemorrhagic fever, dengue shock syndrome**	Case had signs or symptoms as defined in the 1997 WHO Guidelines. Our definitions include:	[31]
Leucopenia	White cell count less than 5.0 × 10^9^ cells/L.	
Plasma Leakage	Case met at least one of following criteria:	[32–36]
	Hemoconcentration: ≥20% increase in hematocrit above the age/sex-specific U.S. mean, or ≥20% hematocrit increase 4–10 days post-illness onset (DPO) compared to sample taken ≤3 DPO with no blood transfusions; Pleural effusion or ascites detected by imaging; Serum albumin <2.5th percentile for age/sex	
**Dengue, dengue with warning signs, severe dengue**	Case had signs or symptoms as outlined in the 2009 WHO Guidelines. Our more specific definition of severe dengue criteria included:	[2]
Severe plasma leakage	Case had plasma leakage (defined above) leading to shock or effusions resulting in acute respiratory distress, respiratory failure or ARDS.	
Severe bleeding	Case had intracranial bleed, or bleeding that resulted in hemodynamic instability requiring fluid replacement and/or blood transfusion.	
Severe organ impairment	Case had acute liver failure, myocarditis, or neurologic impairment necessitating intubation or resulting in death.	
Jaundice	Case had clinically apparent jaundice, or plasma bilirubin greater than 3 mg/dL.	
Acute liver failure (ALF)	Case with no chronic liver disease had acute hepatitis plus hepatic encephalopathy of any grade, jaundice, and new onset coagulopathy defined by international normalized ratio ≥1.5.	[37]
Myocarditis	Case had dyspnea, chest pain, dizziness, or weakness; echocardiographic evidence of global dysfunction; left ventricular ejection fraction <30%, and pericardial effusion or elevated serum troponin T or I.	
Coma	Case had a Glasgow Coma Score of less than 9 and/or was unconscious and unresponsive to painful or verbal stimuli for more than 6 hours.	
**Other Clinical Outcomes**		
Acute hepatitis	Case had serum alanine aminotransferase 10 times the upper limit of normal (ULN) or >400 U/L and no underlying chronic liver disease (e.g., hepatitis C or B, cirrhosis of other or unknown etiology).	
Acute acalculous cholecystitis (AAC)	Case had severe abdominal pain plus two major, or one major and two minor sonographic or CT scan criteria. Gallbladder wall thickening not used as ACC criteria when ascites or hypoalbuminemia present.	[38]
Acute renal failure	Case had at least one of the following criteria:	[39]
	3-fold increase in serum creatinine; 75% decrease in glomerular filtration rate; Serum creatinine ≥4.0 mg/dL with acute increase >0.5 mg/dL; Urine output <0.3 mL/kg/hour in 24 hours or anuria for 12 hours.	
**Medical Complications of Dengue**		
Prolonged shock	Case had hypotension for age for ≥8 hours.	
Metabolic acidosis	Case had an arterial pH <7.35 and bicarbonate <24 mmol/L with a serum bicarbonate within 2 mmol/L, and a normal or low arterial carbon dioxide.	[40]
Fluid overload	Case had periorbital edema, dyspnea, weight gain, or abdominal compartment syndrome.	
Abdominal compartment syndrome	Case had intraabdominal pressure >20 mm Hg with attributable organ failure.	[41]
Acute hypoxemic respiratory failure	Case had an arterial oxygen <60 mm Hg and normal or low arterial carbon dioxide level while on oxygen.	
Acute respiratory distress syndrome (ARDS)	Case met criteria outlined in American-European Consensus Conference definition.	[42]
Healthcare-associated infections (HAI)	Case had infection that became clinically evident >48 hours after hospitalization.	[43]
Disseminated intravascular coagulation (DIC)	Case had a DIC score of ≥5 that accounted for platelet count, D-dimer, PT, and fibrinogen.	[44]

### Data Analysis

Frequencies were calculated for demographic, clinical and laboratory features of fatal laboratory-positive dengue cases. Rates of fatal laboratory-positive dengue cases per 100,000 Puerto Rico population were calculated by age group, sex, and municipality using US Census data [45]. Incidence rate ratios (IRR) were calculated to compare females to males. Case fatality rates (CFR) were calculated by dividing the number of fatal laboratory-positive dengue cases by PDSS laboratory-positive dengue cases. Statistical differences in proportions were tested by Chi-square or Fisher's exact tests. Differences between municipality-specific fatal laboratory-positive dengue cases and PDSS laboratory-positive dengue cases were examined by calculation of Pearson correlation coefficients. A geographically weighted regression model was used to determine if the number of fatal cases differed from the expected based on PDSS laboratory-positive dengue cases in the municipality and neighboring municipalities. Data analyses were conducted using STATA (Stata Corporation, College Station, TX) and ArcGIS (Environmental Systems Research Institute, Redlands, CA); maps were created using ArcMap.

## Results

### Identification of Fatal Cases and Diagnostic Testing

During 2010–2012, a total of 311 fatalities following a dengue-like AFI were detected by EFASS and 40,881 suspected dengue cases were reported to PDSS, of which 17,929 (44%) were dengue laboratory-positive. Of all fatalities detected, 146 (47%) were identified and reported by PRIFS pathologists, 93 (30%) by death certificate review, 50 (16%) by hospital epidemiologists, 15 (5%) by PDSS, four (1%) by NNDSS, and three (1%) by chart review as part of another study. Serum and tissue specimens were available for 148 (48%) cases, serum alone for 138 (44%) cases, tissue alone for 16 (5%) cases, and 9 (3%) cases had no diagnostic specimens. Of the 164 cases (53%) with tissue, one case was not tested because of sample quality. Of evaluable cases, 159 (98%) had liver, 156 (96%) lung, 155 (95%) kidney, 142 (87%) spleen, 98 (60%) lymph nodes, and 66 (41%) intestine. A nasopharyngeal swab was submitted for 27 (9%) cases.

A pathogen was identified in 120 (40%) of 302 cases with a diagnostic specimen. A pathogen was more likely to be identified in cases with tissue specimens than in those without (69% versus 45% respectively, *P* <0.0001). Overall, 58 (19%) fatal cases were dengue laboratory-positive, 167 (54%) were dengue laboratory-negative, and 77 (25%) were dengue laboratory-indeterminate. Other pathogens identified included: *Leptospira* spp. in 34 (11%) cases (32 confirmed, two probable); *Staphylococcus spp*. in nine (3%) cases; *Streptococcus spp*. in nine (3%) cases; influenza A virus in three (1%) cases; and one case each with *Neisseria meningitidis*, *Burkholderia pseudomallei*, *Proteus spp*., *Clostridium perfringens*, *Cryptococcus neoformans*, *Klebsiella pneumoniae*, and an unidentified Gram-positive coccus.

### Characteristics of Fatal Laboratory-Positive Dengue Cases

Of the 58 fatal laboratory-positive dengue cases, 53 (91%) were DENV RT-PCR positive in tissue, serum or both; four (7%) were anti-DENV IgM positive in a single serum specimen; and one (2%) demonstrated anti-DENV IgM seroconversion in paired specimens (Table 2). Autopsies were performed on 26 (45%) of the 58 fatal laboratory-positive dengue cases and DENV was most commonly identified by IHC or RT-PCR in liver (18/26, 69%), lung (15/22, 68%), and kidney (9/24, 38%) tissue. Of 43 cases with rRT-PCR positive serum specimen, 26 (60%) were DENV-1, 16 (37%) DENV-4, and one (2%) DENV-2; similar to DENV-type distribution in PDSS during the same time period [18]. Among the 36 fatal laboratory-positive dengue cases with an available acute serum specimen, 30 (83%) had a secondary DENV infection and 6 (17%) had primary infection. Five of the DENV RT-PCR positive cases had co-infection with another pathogen, including *Leptospira* species (4 cases) [46] and *Streptococcus* species (1 case).

**Table 2:** Diagnostic laboratory results for fatal laboratory-positive dengue cases detected by the Enhanced Fatal Acute Febrile Illness Surveillance System, Puerto Rico 2010–2012.

Diagnostic Result	2010–2012 (N = 58)		2010 (N = 39)		2011 (N = 6)		2012 (N = 13)	
	No.	(%)	No.	(%)	No.	(%)	No.	(%)
Tissue RT-PCR and IHC positive, and serum RT-PCR positive with or without IgM positive	13	22.4	11	28.2	0	0.0	2	15.4
Tissue RT-PCR positive with or without IHC positive only	10	17.2	8*	20.5	0	0.0	2	15.4
Tissue IHC positive and serum RT-PCR and IgM positive	1	1.7	0	0.0	0	0.0	1	7.7
Serum RT-PCR positive with or without IgM positive only	29	50.0	18	46.2	6^†^	100	5^‡^	38.5
Seroconversion by IgM	1	1.7	1	2.6	0	0.0	0	0.0
Serum IgM positive only	4	6.9	1	2.6	0	0.0	3	23.1

* Three were dual infections; two had DENV and *Leptospira spp*. bacteria identified and one had DENV and *Streptococcus pneumonia* identified in tissue.

^†^ One dual infection with DENV and *Leptospira spp*. bacteria identified.

^‡^ One dual infection with DENV and *Leptospira spp*. bacteria identified.

### Epidemiology of Fatal Laboratory-Positive Dengue Cases

**Fig 1:**
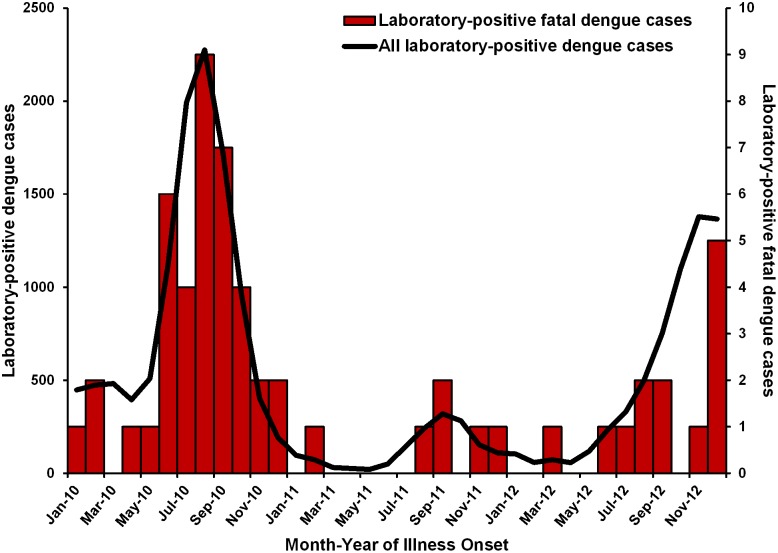
Number of laboratory-positive dengue cases reported to the Passive Dengue Surveillance System and fatal laboratory-positive dengue cases detected by the Enhanced Fatal AFI Surveillance System by month of illness onset, Puerto Rico 2010–2012.

Fatal laboratory-positive dengue cases occurred in the months with increased PDSS dengue reporting (Fig 1). The dengue mortality rate was 1.05 per 100,000 population in 2010, 0.16 in 2011 and 0.36 in 2012; the 3-year average mortality rate was 0.52 dengue deaths per 100,000. The overall CFR for the three-year period was 0.32% and varied from 0.38% during the 2010 epidemic [18],; to 0.39% in 2011, a non-epidemic year, to 0.22% in 2012, an epidemic year.

The median age of fatal laboratory-positive dengue case-patients was 46 years (range: 5 months–89 years). Six (10%) case-patients were <20 years old, 39 (67%) 20–64 years old, and 13 (22%) ≥65 years old. EFASS case-patients were significantly older than laboratory-positive dengue case-patients reported to PDSS (median 46 vs. 18 years, *P <* 0.001) and a higher proportion of fatal laboratory-positive dengue case-patients were adults (90% vs. 49%, respectively, *P <* 0.001).

The majority of fatal laboratory-positive dengue case-patients were female (Table 3), and were significantly more likely to be female than laboratory-positive dengue cases reported to PDSS during the same period (59% vs. 45%, P <0.05). In 2010, rates of fatal laboratory-positive dengue were 1.3 times higher among females than males; 1.19 and 0.90 cases per 100,000 population, respectively (female-to-male IRR = 1.3; 95% confidence interval [CI] = 0.70–2.50, *P* = 0.20). Slightly less than half (27, 47%) of fatal laboratory-positive dengue case-patients were obese, similar to that reported in Puerto Rico [47]. Most fatal laboratory-positive dengue case-patients had more than one pre-existing medical condition (35, 60%). The most common included: diabetes mellitus (23, 40%), asthma (13, 22%), cardiovascular disease (9, 16%), thyroid disease (8, 14%), psychiatric disease (8, 14%), and rheumatologic conditions (8, 14%).

**Table 3:** Characteristics of all fatal laboratory-positive dengue case-patients detected by the Enhanced Fatal AFI Surveillance System, Puerto Rico, 2010–2012.

	Lab-positive dengue cases (n = 58)	Children and adolescents (n = 6)	Adults ≥ 20 years old	
			Dengue only (n = 47)	DENV co-infections (n = 5)
**Demographics, no. (%)**				
Female	34 (59)	5 (83)	29 (62)	0 (0)
Born in Puerto Rico	53 (91)	5 (83)	44 (94)	4 (80)
**Medical history, no. (%)**				
Obese	27 (47)	1 (17)	22 (47)	4 (80)
No chronic disease	10 (17)	3 (50)	7 (15)	0 (0)
One chronic disease	13 (22)	2 (33)	10 (21)	1 (20)
More than one chronic disease	35 (60)	1 (17)	30 (64)	4 (80)
Diabetes	23 (40)	0 (0)	21 (45)	2 (40)
Asthma	13 (22)	2 (33)	10 (21)	1 (20)
Cardiovascular disease	9 (16)	0 (0)	8 (17)	1 (20)
Psychiatric disease	8 (14)	0 (0)	7 (15)	1 (20)
Thyroid disease	8 (14)	0 (0)	8 (17)	0 (0)
Rheumatologic condition	8 (14)	0 (0)	7 (15)	1 (20)
Neurologic disease	6 (10)	0 (0)	6 (13)	0 (0)
Gastrointestinal disease	5 (9)	1(17)	4 (9)	0 (0)

**Fig 2:**
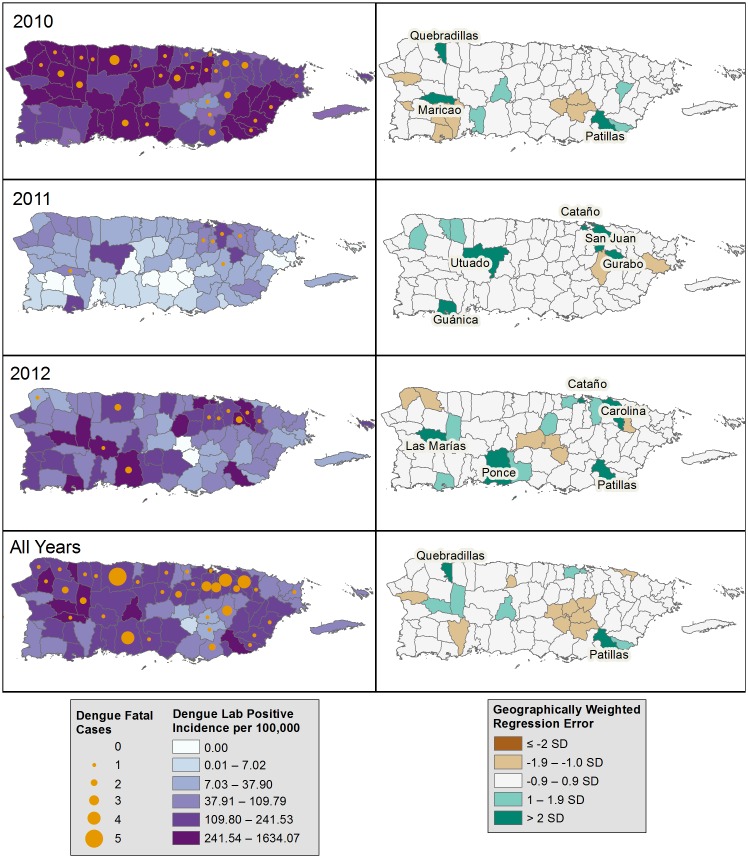
Incidence of laboratory-positive dengue, and observed and expected number of fatal laboratory-positive dengue cases by municipality of residence and year, Puerto Rico, 2010–2012. Left panels: Incidence per 100,000 population of non-fatal, laboratory-positive dengue cases reported to the Passive Dengue Surveillance System, and number of fatal laboratory-positive dengue cases identified by the Enhanced Fatal AFI Surveillance System. Right panels: the standard deviation (SD) of the standard residuals are displayed. Differences >2 SD denotes significantly fewer than expected fatal laboratory-positive dengue cases, while ≤2 SD denotes significantly more than expected fatal laboratory-positive dengue cases.

Fatal laboratory-positive dengue case-patients were residents of 35 of the 78 Puerto Rico municipalities, and most (53, 91%) were born in Puerto Rico (Table 3). Fatal laboratory-positive dengue rates were highest in 2010 in Maunabo (0.82 per 10,000 residents), Maricao in 2011 (1.59), and Adjuntas in 2012 (0.51). In all years, there was a positive correlation between the number of PDSS laboratory-positive dengue cases and the number of fatal laboratory-positive dengue cases in a municipality (R = 0.56, 0.59, 0.62, and 0.73 in 2010, 2011, 2012, and overall, respectively) (Fig 2). The number of fatal laboratory-positive dengue cases detected was no more than expected based on municipality-specific, laboratory-positive dengue incidence rates. However, there were fewer than expected fatal laboratory-positive dengue cases detected in 11 of 78 municipalities in individual years. Only Quebradillas and Patillas had fewer than expected fatalities in all years.

### Healthcare Seeking Behavior of Fatal Laboratory-Positive Dengue Case-Patients

All 58 fatal laboratory-positive dengue case-patients sought care at least once during their illness; 25 (43%) had two healthcare visits, and seven (12%) had three or more visits. The median interval between fever onset and arrival at the first visit was 3.5 days (range: 0–8.5 days). Of the 49 (84%) cases with a medical record for review, at the first visit, most (71%) had dengue fever, while some had severe dengue (35%) or DHF (18%) (Table 4); the majority (67%) had warning signs for severe dengue, the most common being persistent vomiting (21 of 33, 64%), abdominal pain (15, 45%), and mucosal bleeding (14, 42%). The leading diagnoses during the first visit were dengue, viral syndrome, gastroenteritis, and urinary tract infection. At the first visit, 17 (35%) were admitted to the hospital, 7 (14%) were transferred to another hospital, 7 (14%) died in the ED, and 18 (37%) were discharged home. Of those transferred to another hospital, six (86%) were admitted and died in the ICU, and one died in the ED.

**Table 4:** Clinical features and outcomes for fatal laboratory-positive dengue case-patients detected by the Enhanced Fatal AFI Surveillance System, Puerto Rico, 2010–2012.

	First outpatient healthcare visit*		At time of death in the hospital	
	N = 49		N = 55	
Days post onset, median (range)	3.5	(0–8.5)	4.5	(0.5–13.0)
No. prior visits, median (range)	NA		2	(1–5)
**Clinical diagnosis,**^†^ **no. (%)**				
Dengue	25	(51.0)	34	(61.8)
Viral syndrome	7	(14.3)	2	(3.6)
Gastroenteritis	6	(12.2)	3	(5.5)
Urinary tract infection	3	(6.1)	1	(1.8)
Leptospirosis	2	(4.1)	4	(7.3)
Multi-organ failure	2	(4.1)	4	(7.3)
Pancytopenia	1	(2.0)	3	(5.5)
Dengue and leptospirosis	1	(2.0)	1	(1.8)
Respiratory tract infection	1	(2.0)	0	---
Dehydration	1	(2.0)	0	---
Meningitis with shock	0	---	2	(3.6)
Myocarditis	0	---	1	(1.8)
**Signs and symptoms, no. (%)**				
Fever measured at facility	28	(57.1)	32	(58.2)
Headache	21	(42.9)	27	(49.1)
Eye pain	6	(12.2)	10	(18.2)
Muscle pain	31	(63.3)	33	(60.0)
Joint pain	14	(28.6)	23	(41.8)
Bone pain	9	(18.4)	15	(27.3)
Rash	9	(18.4)	19	(34.6)
Any bleeding manifestation	14	(28.6)	47	(85.5)
Vomiting	21	(42.9)	34	(61.8)
Abdominal pain	15	(30.6)	31	(56.4)
Diarrhea	12	(24.5)	26	(47.3)
Cough	7	(14.3)	21	(38.2)
Sore throat	6	(12.2)	6	(10.9)
**Clinical laboratory findings**				
White blood cells (10^9^/L), median (range)	4.6	(0.94–19.1)	4.9	(0.94–18.6)
Leukopenia, no. (%)	25	(51.0)	29	(52.7)
Platelet count (10^9^/L), median (range)	79	(8–367)	55	(7–269)
Thrombocytopenia, no. (%)	29	(59.2)	46	(83.6)
Hemoconcentrated, no. (%)	11	(22.5)	8	(14.6)
Aspartate aminotransferases (U/L), median (range)	212	(29–5,733)	284	(22–15,481)
Alanine aminotransferases (U/L), median (range)	187	(23–22,046)	176	(23–22,046)
Aminotransferases ≥1000 U/L, no. (%)	9	(18.4)	18	(32.7)
Serum sodium ≤125 mEq/L, no. (%)	2	(4.1)	8	(14.6)
**Clinical syndrome, no. (%)**				
Dengue fever	35	(71.4)	47	(85.5)
Dengue hemorrhagic fever	9	(18.4)	27	(49.1)
Dengue shock syndrome	5	(10.2)	19	(34.6)
Had warning sign(s)	33	(67.3)	53	(96.4)
Severe dengue	17	(34.7)	45	(81.8)
**Disposition, no. (%)**				
Discharged to home	18	(37.0)	NA	---
Transferred to another healthcare facility	7	(14.0)	NA	---
Died in Emergency Department	7	(14.0)	12	(22.0)
Died on inpatient ward	2	(4.0)	10	(18.0)
Died in intensive care unit	15	(31.0)	33	(60.0)

*First healthcare visit may have been at a private clinic or a hospital emergency department. Data does not include that collected after admission to the hospital or transfer to another hospital.

^**†**^ Case-patients could have had more than one diagnosis listed as a discharge, admission or transfer diagnosis.

Among the 18 case-patients discharged home after their first visit, seven (39%) had at least one recorded warning sign, including compensated shock or hemorrhagic manifestations. Median age was 36.6 years (range 0.6–74.9), 14 (78%) were female, and 16 (89%) had at least one chronic medical condition. Two died at home after being discharged; diagnoses of dengue and moderate dehydration, and acute gastroenteritis. The remaining 16 returned to a hospital on average 2 days (range 0.0–3.5 days) after discharge. Nine (56%) died during their second visit, four (25%) were transferred to another facility, and three (19%) were again discharged home.

Overall, the median interval between illness onset and death was 7.1 days (range: 1.2–28.4 days); in six (10%) cases the interval exceeded 14 days. Most (43, 74%) case-patients died as an inpatient in a hospital; however, 12 (21%) died in the Emergency Department prior to hospital admission, and three (5%) died at home: one after a 2-day hospitalization, and two after being seen in an ED (Table 4). Of the 43 case-patients who died after being admitted, 33 (57%) died in the intensive care unit, and 10 (17%) died in an inpatient ward. The median interval from hospital admission to death was 1.9 days (range: 0.1–28.8 days).

Only 25 of 58 (43%) fatal laboratory-positive dengue case-patients had dengue, DHF, DSS, or dengue-like syndrome listed as primary (17 cases) or contributing (8 cases) cause of death on their death certificate. The five most common primary causes of death included dengue (29%), viral syndrome (16%), cardiorespiratory failure (12%), sepsis (9%), and thrombocytopenia (5%).

### Clinical Outcomes Among Fatal Laboratory-Positive Dengue Case-Patients

Most of the 55 laboratory-positive dengue case-patients who died in hospital had signs and symptoms consistent with dengue (86%) or severe dengue (82%), but only 62% had that clinical diagnosis (Table 4). Of those who met criteria for severe dengue by the time of death, 38 of 45 (84%) had severe plasma leakage, 25 (56%) had severe bleeding, and 22 (49%) had severe organ impairment. Of note, hemoconcentration was documented in only a few case-patients even when hematocrits were performed over time. However, 34 (62%) case-patients who died in hospital had an effusion and most (62%) case-patients with effusions had acute respiratory failure or ARDS (Table 5).

**Table 5:** Demographic characteristics, medical history and clinical outcomes for fatal laboratory-positive dengue case-patients detected by the Enhanced Fatal AFI Surveillance System who died in a hospital, Puerto Rico, 2010–2012.

			Adults ≥20 years old	
	All cases (n = 55)	Children and adolescents (n = 6)	Dengue only (n = 44)	Co-infections* (n = 5)
Female, no. (%)	32 (58.2)	5 (83.3)	27 (61.4)	0 (0.0)
Obese, no. (%)	24 (43.6)	1 (16.7)	19 (43.2)	4 (80.0)
No chronic disease, no. (%)	10 (18.2)	3 (50.0)	7 (15.9)	0 (0.0)
One chronic disease, no. (%)	12 (21.8)	2 (33.3)	9 (20.5)	1 (20.0)
More than one chronic disease, no. (%)	33 (60.0)	1 (16.7)	28 (63.6)	4 (80.0)
**Clinical outcomes**				
Days post-illness onset at admission, median (range)	4.5 (0.5–13.0)	4.6 (0.5–8.9)	4.5 (0.5–13.0)	4.7 (3.6–7.2)
Length of hospital stay, median (range)	2.7 (1.1–29.2)	8.6 (8.6–8.6)	2.7 (1.1–29.2)	2.3 (2.3–2.3)
Admitted to ICU, no. (%)	37 (67.3)	4 (66.7)	29 (65.9)	4 (80.0)
ICU length of stay, median (range)	1.1 (0.1–25.8)	2.5 (0.4–4.9)	1.1 (0.1–25.8)	1.1 (0.7–5.2)
**Clinical laboratory findings**^†^				
Leukopenia, no. (%)	29 (52.7)	3 (50.0)	24 (54.6)	2 (40.0)
Thrombocytopenia, no. (%)	46 (83.6)	4 (66.7)	37 (84.1)	5 (100.0)
Hematocrit increase by ≥ 20%, no. (%)	7 (12.7)	0 (0.0)	7 (15.9)	0 (0.0)
Hemoconcentration by age, no. (%)	1 (1.8)	0 (0.0)	1 (2.3)	0 (0.0)
Days post onset of max HCT, median (range)	4 (0–13)	4 (0–9)	4 (1–13)	4 (3–7)
**Effusion, no. (%)**^‡^				
Any effusion	34 (61.8)	6 (100.0)	26 (59.1)	2 (40.0)
Pleural effusion	24 (43.6)	4 (66.7)	19 (43.2)	1 (20.0)
Ascites	14 (25.5)	4 (66.7)	10 (22.7)	0 (0.0)
Pericardial effusion	9 (16.4)	1 (16.7)	7 (15.9)	1 (20.0)
Effusion with respiratory failure or ARDS	21 (38.2)	4 (66.7)	16 (36.4)	1 (20.0)
**Bleeding Manifestation, no. (%)**				
Any bleeding**	47 (85.5)	6 (100.0)	38 (86.4)	3 (60.0)
Severe bleed	25 (45.5)	4 (66.7)	18 (40.9)	3 (60.0)
**Other Clinical Features, no. (%)**				
Acute hepatitis	42 (76.4)	5 (83.3)	32 (72.7)	5 (100.0)
Acute liver failure	5 (9.1)	2 (33.3)	3 (6.8)	0 (0.0)
Cholecystitis	5 (9.1)	1 (16.7)	3 (6.8)	1 (20.0)
Myocarditis	2 (3.6)	0 (0.0)	2 (4.6)	0 (0.0)
Acute renal failure	10 (18.2)	1(16.7)	7 (15.9)	2 (40.0)
**Medical complications, no. (%)**				
Prolonged shock	29 (52.7)	2 (33.3)	23 (52.3)	4 (80.0)
Metabolic acidosis	36 (65.5)	5 (83.3)	27 (61.4)	4 (80.0)
Fluid overload	17 (30.9)	4 (66.7)	11 (25.0)	2 (40.0)
Abdominal compartment syndrome	2 (3.6)	2 (33.3)	0 (0.0)	0 (0.0)
Acute respiratory failure	30 (54.6)	5 (83.3)	21 (47.7)	4 (80.0)
Acute respiratory distress syndrome	11 (20.0)	4 (66.7)	5 (11.4)	2 (40.0)
Coma	10 (18.2)	2 (33.3)	8 (18.2)	0 (0.0)
Seizure	8 (14.6)	1 (16.7)	7 (15.9)	0 (0.0
Hospital acquired infection	16 (29.1)	2 (33.3)	11 (25.0)	3 (60.0)
Disseminated intravascular coagulation	5 (9.1)	2 (33.3)	3 (6.8)	0 (0.0)
**Treatment given, no. (%)**				
Intravenous colloid	16 (29.1)	3 (50.0)	11 (25.0)	2 (40.0)
Blood transfusion	13 (23.6)	2 (33.3)	10 (22.7)	1 (20.0)
Platelet transfusion	26 (47.3)	2 (33.3)	22 (50.0)	2 (40.0)
Fresh frozen plasma	12 (21.8)	2 (3.33)	9 (20.5)	1 (20.0)
Inotropes	38 (69.1)	4 (66.7)	30 (68.2)	4 (80.0)
Diuretics	22 (40.0)	3 (50.0)	16 (36.4)	3 (60.0)
Corticosteroid	25 (45.5)	0 (0.0)	22 (50.0)	3 (60.0)

* Co-infections include: Four DENV/*Leptospira spp*. bacteria, and one DENV/*Streptococcus pneumonia*.

^**†**^ Number and percent presented unless otherwise stated.

^**‡**^ Most (44, 80%) of the 55 case-patients who died in hospital had a chest x-ray and/or an ultrasound done. Several case-patients (30, 55%) had at least one other imaging study done including an echocardiogram (17 done) and/or a computed tomography (CT) scan (2 abdominal and 23 brain CT scans done).

** Any bleeding was defined by the presence of any of the following: petechiae, purpura, ecchymosis, epistaxis, gingival bleeding, hematuria, menorrhagia, hemoptysis, hematemesis, melena, or an intracranial bleed.

The majority of case-patients who died in a hospital (86%) were bleeding, and 25 (46%) had severe bleeding: 19 (35%) gastrointestinal, 14 (26%) pulmonary, six (11%) vaginal, and six (11%) intracranial (Table 5). Of those with severe bleeding, 14 (56%) received a platelet transfusion while 11 (44%) received a blood transfusion; blood transfusion was more likely to occur with increased hospital stay (median 5.2 vs. 1.3 days, respectively; *P* <0.001). Of the 26 case-patients who received a platelet transfusion, three (12%) had no recorded bleeding. The best predictor of platelet transfusion was low platelet count [median 13,000 (range: 7,000–37,000) vs. 55,000 (range: 8,000–224,000) cells/mm^3^, recipients versus non-recipients, respectively; *P* <0.001].

Other severe clinical outcomes among the 55 laboratory-positive dengue case-patients who died in hospital included acute hepatitis (76%), acute renal failure (18%), cholecystitis (9%), acute liver failure (9%), myocarditis (4%), metabolic acidosis (66%), prolonged shock (53%), and acute respiratory failure (55%) (Table 5). All received intravenous crystalloids, 40% received a diuretic, and 29% received intravenous colloids. Twenty-five (45%; all adults) received intravenous corticosteroids. Those given steroids had a lower median platelet count than those not given steroids (16,000 vs. 47,000 cells/mm^3^; respectively, *P* <0.01), and were three times more likely to have a hospital acquired infection (75% vs. 25%, *P* <0.05).

## Discussion

Enhanced surveillance for dengue deaths showed the majority were not reported to the standard dengue surveillance system and most did not have “dengue” coded on the death certificate. Identification of these unrecognized deaths resulted in a 2 to 3-fold higher dengue mortality rate than previously reported [14, 17, 20, 21, 48, 49]. EFASS demonstrated the importance of appropriate diagnostic testing of tissue and serum to make the correct diagnosis in deaths from a dengue-like acute febrile illness. In addition, EFASS showed its ability to identify unrecognized deaths from other pathogens of public health importance.

The EFASS estimated age-specific annual dengue mortality rates were comparable to those from other infectious diseases in the US, including influenza [50, 51]. However, in contrast to influenza, most dengue deaths occurred among adults 19–64 years of age. The estimated average annual influenza-associated US death rate is 2.4 per 100,000 residents (range: 0.4–5.1). In most years, 88% of these deaths are among persons aged ≥65 years [51, 52]; 17.0 deaths per 100,000 (range: 2.4–36.7). Influenza death rates among persons <19 years and 19–64 years are 0.1 (range: 0.1–0.3) and 0.4 (range: 0.1–0.8) per 100,000, respectively. In comparison, EFASS estimated that dengue mortality in 2010 was 0.42, 1.17, and 1.66 per 100,000 persons aged <19 years, 19–64 years and ≥65 years, respectively.

Most fatal laboratory-positive dengue case-patients appeared to have timely access to healthcare. However, many (~40%) were sent home after their first ED visit with warning signs of severe dengue. Although the majority sought care again within 48 hours, two died at home. Most case-patients who died in a hospital had severe plasma leakage, severe bleeding, or both, and most received inotropes and half received a platelet transfusion. Although bleeding was present in the majority who received platelets, half of those with severe bleeding did not receive red blood cells. A large proportion of case-patients received corticosteroids, which are not considered of benefit in dengue [2, 53]. As reported by others, we found an increased risk of hospital-acquired infections in these patients [54, 55].

Dengue deaths often occur among patients with comorbidities [14, 19]. Nearly half of case-patients were obese and over half had more than one chronic medical condition; prevalences similar to those found in the Puerto Rican adult population [47, 56], with the exception of diabetes and asthma. The prevalence of diabetes in case-patients was nearly four times that of the adult population, and asthma was twice as prevalent. Adult diabetics have been over-represented in other fatal case series [19, 57], and a recent meta-analysis found diabetes was associated with increased risk of severe dengue [58]. As many endemic areas have reported a substantial proportion of dengue cases in adults, healthcare providers should be attentive to dengue patients with these comorbidities [2].

Some patients developed acute liver or renal failure or had atypical presentations [19, 57, 59, 60]. Acute renal failure (ARF) affected ~20% of case-patients though none had pre-existing renal disease and 80% were non-elderly (median age 49 years). However, dengue patients with severe dengue, diabetes or secondary infections are known to be at risk for developing acute kidney injury [61]. Six of the ten ARF cases had at least one risk factor and two were co-infected with *Leptospira spp*. One of the four ARF case-patients without risk factors was an infant with abdominal compartment syndrome and multiple organ dysfunction.

While more sensitive than PDSS, EFASS may not have detected all fatal laboratory-positive dengue cases. For example, a few rural municipalities had fewer deaths than expected. In the case of Patillas, this may have been due to higher dengue case-reporting to PDSS because of an enhanced dengue surveillance project conducted prior to EFASS [62]. Alternatively, individuals in rural municipalities who died at home and were not known to have an AFI would not have been identified. These factors may have led to lower case ascertainment and estimated dengue mortality. Although we increased the proportion of suspect cases with an etiologic diagnosis by obtaining tissue and convalescent serum specimens, about one quarter of cases were dengue laboratory-indeterminate, and were not counted as fatal laboratory-positive dengue cases even if dengue was listed on their death certificate. Hence, our final dengue mortality estimate should be considered conservative.

EFASS demonstrated the feasibility and importance of enhanced surveillance for dengue deaths, and found a previously unrecognized high dengue mortality in Puerto Rico that was higher than rates observed in other dengue endemic regions during this time period [9–14]. Establishment of EFASS-like systems in selected dengue endemic countries would go a long way towards obtaining robust estimates of the global burden of deaths due to dengue, and identify areas for improvement in clinical care of patients with severe dengue.

## Supporting Information

S1 Appendix ICD-9 codes used to review death certificates and medical records to identify individuals that died following a dengue-like acute febrile illness. (PDF)

S2 Appendix Surgical Pathology and Autopsy Report (SPAR) Form used by forensic pathologists to document autopsy findings from individuals that died following a dengue-like acute febrile illness. (PDF)

S1 Checklist STROBE Checklist. (DOCX)
