# Supplementary material for: Enhanced Surveillance for Fatal Dengue-Like Acute Febrile Illness in Puerto Rico, 2010-2012
Source: PLoS Negl Trop Dis. 2016 Oct 11;10(10):e0005025. doi: 10.1371/journal.pntd.0005025 (PMC5058557; doi:10.1371/journal.pntd.0005025)
Supplement: S1 Appendix — (PDF) [file pntd.0005025.s001.pdf]

ICD-10 Code	ICD-10 Code Title	ICD-9 Code	ICD-9 Code Title
**Clinical Sepsis, Viral Syndromes, Dengue Fever and Dengue Hemorrhagic Fever**			
A41.9	Sepsis, unspecified	038.9	Unspecified septicemia
A41.9	Sepsis	995.91	Sepsis
A90	Dengue fever	061	Dengue
A91	Dengue hemorrhagic fever	065.4	Mosquito-borne hemorrhagic fever
A92.9	Mosquito-borne viral fever, unspecified	066.9	Arthropod-borne viral disease, unspecified
A99	Unspecified viral hemorrhagic fever	065.9	Arthropod-borne hemorrhagic fever, unspecified
B34.9	Viral infection, unspecified. Includes: Viremia, not otherwise specified (NOS)	790.8	Unspecified viremia
R65.20	Severe sepsis	995.92	Severe sepsis
**Shock or Dehydration**			
R57.1	Hypovolemic shock	785.59	Other shock, without trauma
R57.8	Other shock	785.59	Other shock, without trauma
R57.9	Shock unspecified	785.50	Shock, unspecified
R65.21	Severe sepsis with septic shock	785.52	Severe sepsis with septic shock
E86	Dehydration	276.51	Dehydration
**Acidosis**			
E87.2	Acidosis NOS, Lactic acidosis, metabolic, or respiratory acidosis. Excludes: Diabetic acidosis (E10-E13 with ketoacidosis)	276.2	Acidosis
E87.8	Other disorders of electrolyte and fluid balance, not elsewhere classified	276.9	Electrolyte and fluid disorders, not elsewhere classified
**Hemorrhage**			
A27.9	Leptospirosis icterohemorrhagica	100.9	Leptospirosis
K92.2	Gastrointestinal hemorrhage, unspecified	578.9	Hemorrhage of gastrointestinal tract, unspecified
R58.8	Hemorrhage, not classified elsewhere	459.0	Hemorrhage, unspecified
**Thrombocytopenia**			
D69.42	Congenital and hereditary thrombocytopenia purpura	287.33	Congenital and hereditary thrombocytopenia purpura
D69.49	Other primary thrombocytopenia	287.39	Primary thrombocytopenia, other
D69.5	Secondary thrombocytopenia	287.4	Secondary thrombocytopenia
D69.6	Thrombocytopenia, unspecified	287.5	Thrombocytopenia, unspecified
P61.0	Transient neonatal thrombocytopenia	776.1	Transient neonatal thrombocytopenia

ICD-10 Code	ICD-10 Code Title	ICD-9 Code	ICD-9 Code Title
**Encephalitis, Reye’s Syndrome, and Guillain-Barré Syndrome**			
A83.8	Other mosquito-borne viral encephalitis	062.8	Other specified mosquito-borne viral encephalitis
A83.9	Mosquito-borne viral encephalitis, unspecified	062.9	Mosquito-borne viral encephalitis, unspecified arthropod
A85.2	Arthropod-borne viral encephalitis, unspecified	064	Viral encephalitis transmitted by other and unspecified
A85.8	Other specified viral encephalitis	049.8	Other specified non-arthropod-borne viral disease of central nervous system (CNS)
A86.6	Unspecified viral encephalitis	049.9	Unspecified non-arthropod-borne viral disease of CNS
A89.9	Unspecified viral infection of CNS	049.9	Unspecified non-arthropod-borne viral disease of CNS
G61.0	Guillain-Barré syndrome	357.0	Acute infective polyneuritis
G93.7	Reye’s syndrome	331.81	Reye’s syndrome
**Pleural Effusion**			
J90.0	Pleural effusion, not elsewhere classified	511.89<br>511.9	Hemothorax<br>Unspecified pleural effusion
**Ascites**			
R18.8	Other ascites	789.59	Ascites, other
**Sudden Unexplained Deaths**			
R95	Sudden infant death syndrome	798.0	Sudden infant death syndrome
R96	Other sudden death, cause unknown	-----	No translation
R96.0	Instantaneous death	798.1	Instantaneous death
R96.1	Death occurring less than 24 hours from onset of symptoms, not otherwise explained	798.2	Death occurring less than 24 hours from onset of symptoms, not otherwise explained
R98	Unattended death	798.9	Unattended death
R99	Other ill-defined and unspecified causes of mortality	799.9	Other ill-defined and unspecified causes of mortality
