# Supplementary material for: Enhanced Surveillance for Fatal Dengue-Like Acute Febrile Illness in Puerto Rico, 2010-2012
Source: PLoS Negl Trop Dis. 2016 Oct 11;10(10):e0005025. doi: 10.1371/journal.pntd.0005025 (PMC5058557; doi:10.1371/journal.pntd.0005025)
Supplement: S2 Appendix — (PDF) [file pntd.0005025.s002.pdf]

Fatal Case Surveillance for Deaths due to Dengue-like-illness

Surgical Pathology and Autopsy Report (SPAR)

Demographic Information

Full Name: ______________________ Sex: male ☐ female ☐

Paternal Last *Maternal Last* *First* *M.I.*

Address: ______________________

Urbanization *Barrio or Sector* *Street Address* *Apartment/Unit #*

Municipality *State* *ZIP Code*

Weight ☐ lbs Height ☐ inches Blood type (circle):

A+	B+	AB+	O+
A-	B-	AB-	O-

Date of Death: ______ mm dd yyyy

Time of Death: ______ a.m. p.m.

Place of death (Specify Hospital): ______

Place of birth: ______ Number of years living in PR: ______ years

Autopsy Results

	YES	NO			
Ascites?	☐	☐	If yes, how much?	cc	Describe:
Right pleural effusion?	☐	☐	If yes, how much?	cc	Describe:
Left pleural effusion?	☐	☐	If yes, how much?	cc	Describe:
Pericardial effusion?	☐	☐	If yes, how much?	cc	Describe:
Other effusion?	☐	☐	If yes, how much?	cc	Describe:

Describe location:

	YES	NO			
Pulmonary hemorrhage?	☐	☐	If yes, how much?	cc	Describe:

Describe location:

	YES	NO			
Intestinal hemorrhage?	☐	☐	If yes, how much?	cc	Describe:

Describe location:

	YES	NO			
Other hemorrhage?	☐	☐	If yes, how much?	cc	Describe:

Describe location:

	YES	NO			
Intracranial bleed?	☐	☐	If yes, how much?	cc	Describe:

Describe location:

	YES	NO			
Other significant gross findings?	☐	☐	If yes, how much?	cc	Describe:

Describe location:

Skin Findings

	YES	NO		
Petechiae?	☐	☐	If yes, how many?	Location:
Purpura?	☐	☐	If yes, how many?	Location:

Ecchymosis?	YES ☐	NO ☐	If yes, how many?	Location:
Edema?	YES ☐	NO ☐	If yes, where?	
Skin rash?	YES ☐	NO ☐	If yes, where and describe?	
Jaundice, icteric?	YES ☐	NO ☐	Describe:	
Other skin findings?	YES ☐	NO ☐	Describe:	

Describe location:

Check List for Tissue Collection

Tissue	Collect tissues 1-7 on list below for ALL CASES. Collect multiple 1x2 cm tissue pieces except for 6 and 7. Collect 8-15 as needed.	Check list		
1. Liver	Peritoneal, towards hilum, central **(3 pieces from liver)**	Done:	YES ☐	NO ☐
2. Lung	Base, left peripheral, right peripheral, hilum, tracheobronchial (**5 pieces from lung)**	Done:	YES ☐	NO ☐
3. Kidney	Left (apex), right (apex) **(2 pieces from kidney)**	Done:	YES ☐	NO ☐
4. Spleen	Apex, middle, base **(3 pieces from spleen)**	Done:	YES ☐	NO ☐
5. Lymph node	Specify: mesenteric, mediastinal **(2 from lymph node)**	Done:	YES ☐	NO ☐
6.Bone marrow	Marrow from ribs, place sample in formalin	Done:	YES ☐	NO ☐
7. Skin with rash	Skin from rash effected area (1 piece 3-5 mm or 4mm punch biopsy)	Done:	YES ☐	NO ☐
8. Thymus	Central medulla, a peripheral cortex **(2 pieces from thymus)**	Done:	YES ☐	NO ☐
9. Adrenals	Left, right **(2 from adrenals)**	Done:	YES ☐	NO ☐
10. Cerebellum	Collect tissue if mental status changes	Done:	YES ☐	NO ☐
11. Cerebrum	Collect tissue if mental status changes	Done:	YES ☐	NO ☐
12. Brain stem	Collect tissue if mental status changes	Done:	YES ☐	NO ☐
13. Intestine	Collect if GI bleeding<br>Small, Large **(2 pieces from intestine)**	Done:	YES ☐	NO ☐
14. Heart	Collect if cardiac effusion<br>Left ventricle, right ventricle **(2 pieces from heart)**	Done:	YES ☐	NO ☐
15. Urinary Bladder	Collect if urinary bleeding<br>Wall **(1 piece from urinary bladder)**	Done:	YES ☐	NO ☐

Body Fluid Collection

Body Fluid	Specifications on body fluid sampling technique	Check list		
Whole blood from heart	Two, 10 ml EDTA tubes	Done:	YES ☐	NO ☐
Cerebral spinal fluid	Red top tube, marked CSF	Done:	YES ☐	NO ☐
Effusion fluid from lung	Red top tube, marked pleural effusion	Done:	YES ☐	NO ☐

Signature and mailing address of submitter

Signature:______________________________ Date:__________________

Address: ______________________________ Telephone: _______________
